# Evaluation of Host Protein Biomarkers by ELISA From Whole Lysed Peripheral Blood for Development of Diagnostic Tests for Active Tuberculosis

**DOI:** 10.3389/fimmu.2022.854327

**Published:** 2022-05-20

**Authors:** Harriet N. Garlant, Kalaiarasan Ellappan, Matthew Hewitt, Prem Perumal, Simon Pekeleke, Nadina Wand, Jo Southern, Saka Vinod Kumar, Harish Belgode, Ibrahim Abubakar, Sanjeev Sinha, Seshadri Vasan, Noyal Mariya Joseph, Karen E. Kempsell

**Affiliations:** ^1^Science Group: Research and Evaluation, UK Health Security Agency, Salisbury, United Kingdom; ^2^Department of Microbiology, Jawaharlal Institute of Postgraduate Medical Education and Research, Puducherry, India; ^3^School of Life & Medical Sciences, Mortimer Market Centre, University College London, London, United Kingdom; ^4^Department of Medicine, All India Institute for Medical Sciences, New Delhi, India; ^5^Department of Health Sciences, University of York, York, United Kingdom

**Keywords:** biomarker, protein, ELISA, tuberculosis, diagnostic, assay, diagnosis

## Abstract

Tuberculosis (TB) remains a significant global health crisis and the number one cause of death for an infectious disease. The health consequences in high-burden countries are significant. Barriers to TB control and eradication are in part caused by difficulties in diagnosis. Improvements in diagnosis are required for organisations like the World Health Organisation (WHO) to meet their ambitious target of reducing the incidence of TB by 50% by the year 2025, which has become hard to reach due to the COVID-19 pandemic. Development of new tests for TB are key priorities of the WHO, as defined in their 2014 report for target product profiles (TPPs). Rapid triage and biomarker-based confirmatory tests would greatly enhance the diagnostic capability for identifying and diagnosing TB-infected individuals. Protein-based test methods e.g. lateral flow devices (LFDs) have a significant advantage over other technologies with regard to assay turnaround time (minutes as opposed to hours) field-ability, ease of use by relatively untrained staff and without the need for supporting laboratory infrastructure. Here we evaluate the diagnostic performance of nine biomarkers from our previously published biomarker qPCR validation study; CALCOCO2, CD274, CD52, GBP1, IFIT3, IFITM3, SAMD9L, SNX10 and TMEM49, as protein targets assayed by ELISA. This preliminary evaluation study was conducted to quantify the level of biomarker protein expression across latent, extra-pulmonary or pulmonary TB groups and negative controls, collected across the UK and India, in whole lysed blood samples (WLB). We also investigated associative correlations between the biomarkers and assessed their suitability for ongoing diagnostic test development, using receiver operating characteristic/area under the curve (ROC) analyses, singly and in panel combinations. The top performing single biomarkers for pulmonary TB versus controls were CALCOCO2, SAMD9L, GBP1, IFITM3, IFIT3 and SNX10. TMEM49 was also significantly differentially expressed but downregulated in TB groups. CD52 expression was not highly differentially expressed across most of the groups but may provide additional patient stratification information and some limited use for incipient latent TB infection. These show therefore great potential for diagnostic test development either in minimal configuration panels for rapid triage or more complex formulations to capture the diversity of disease presentations.

## 1 Introduction

Tuberculosis (TB) continues to be a leading cause of morbidity and mortality worldwide, accounting for the deaths of an estimated 1.5 million people each year, including 214,000 among HIV positive people in 2020 (1). This figure is comparable to the 1.8 million deaths due to COVID-19 alone during the current pandemic which is also contributing to TB resurgence (2, 3). India has the highest global TB burden, accounting for one fifth of the TB incidence worldwide, with 40% of the total Indian population estimated to be infected with TB (4). In the UK, most TB cases are concentrated in large urban centres where the incidence can be greater than 1/20,000 - one of the highest rates of any Western country (5). Most TB cases (72.8%) occur among non-UK born individuals, who have emigrated from countries with a high burden of endemic TB (6). TB presents predominantly as the pulmonary manifestation (PTB) in the lung, but can affect any organ or tissue, manifesting as a myriad of presentations termed ‘extra-pulmonary’ tuberculosis [EPTB (7–10)]. This form of the disease is hard to diagnose using conventional methods, as is quiescent or latent TB infection [LTBI (4, 11, 12)]. Therefore, despite ongoing investment in research and development for new diagnostics and therapeutics, TB eradication has proved challenging (13).

Ending the Global TB epidemic by 2030 is a priority in the newly adopted WHO Sustainable Development Goals (14). Rapid non-sputum-based tests for detecting TB and community-based triage tests for identifying suspected TB infected individuals are key priorities for the World Health Organization target product profiles (TPPs), set out in their 2014 report (15). Additionally, a test able to diagnose LTBI or incipient active TB (iATB) would greatly improve early diagnosis, assist with patient management programs, reduce disease dissemination and the current socio-economic disease burden (16–18). There is therefore clearly an urgent need for the development of rapid, inexpensive and accurate tests for diagnosis of TB particularly in the point of care (POC) and remote settings (19–23). A biomarker protein-based, non-sputum diagnostic test such as an LFD would fulfil these criteria. The development of multiplex LFDs able to detect large numbers of analytes simultaneously has recently been achieved (24), however LFDs with two to three analytes are more commonly reported (25). Therefore, in order to configure LFDs for TB triage diagnosis, minimal analyte configurations are most likely required. Multianalyte configurations could be useful in more complex assay formats, e.g. ELISA in the laboratory setting and amenable to confirmatory test application.

Host immune biomarkers which are specifically and differentially expressed during exposure or infection have become an attractive prospect for the detection and diagnosis of TB (11). There have now been a large number of studies conducted to identify and validate high performing, TB disease-specific biomarkers as RNA targets, which have shown potential for use in identifying individuals with exposure or infection with MTB in all its varying presentations, viz. PTB, EPTB and LTBI. Diagnostic and prognostic biomarkers, which are predictive of adequate responsiveness to treatment and of risk of developing active TB in LTBI are also major goals for TB investigators and clinicians (3). However, nucleic acid targets are more amenable to laboratory-based devices and the methodologies have relatively long processing and turnaround times, even if they can be rapidly adapted for new or emerging pathogens, strains and variants (26, 27).

There have been fewer reports of biomarker indicators assayed as proteins for TB, but some studies have been conducted using saliva, serum and plasma samples (28–34). Protein biomarkers are predicted to be most useful target for simple, fast and cost-effective ‘point of care’ tests (35) required to improve diagnosis rates in resource-limited settings (36, 37). The upfront work required to develop protein assay reagents is significantly more laborious and time consuming than for nucleic acid targets (24). The rewards with respect to cost and assay turnaround time are significantly higher compared to nucleic acid-based methodologies (38–41).

Here we present a preliminary evaluation study to assess the diagnostic performance of nine of our previously identified, published mRNA biomarkers (13) as protein targets; CALCOCO2, CD274, CD52, GBP1, IFIT3, IFITM3, SAMD9L, SNX10 and TMEM49. Expression was assessed using commercial ELISA assays, these were then analysed individually and in combination panels to establish best candidates for future progression in developing protein-based TB diagnostic POC tests. Expression correlation matrix analyses were also conducted to gain an understanding of their inter-target relationships/influences, biological functional significance and potential for TB disease sub-type stratification.

## 2 Methodology

### 2.1 Sample Collection

**Figure 1:**
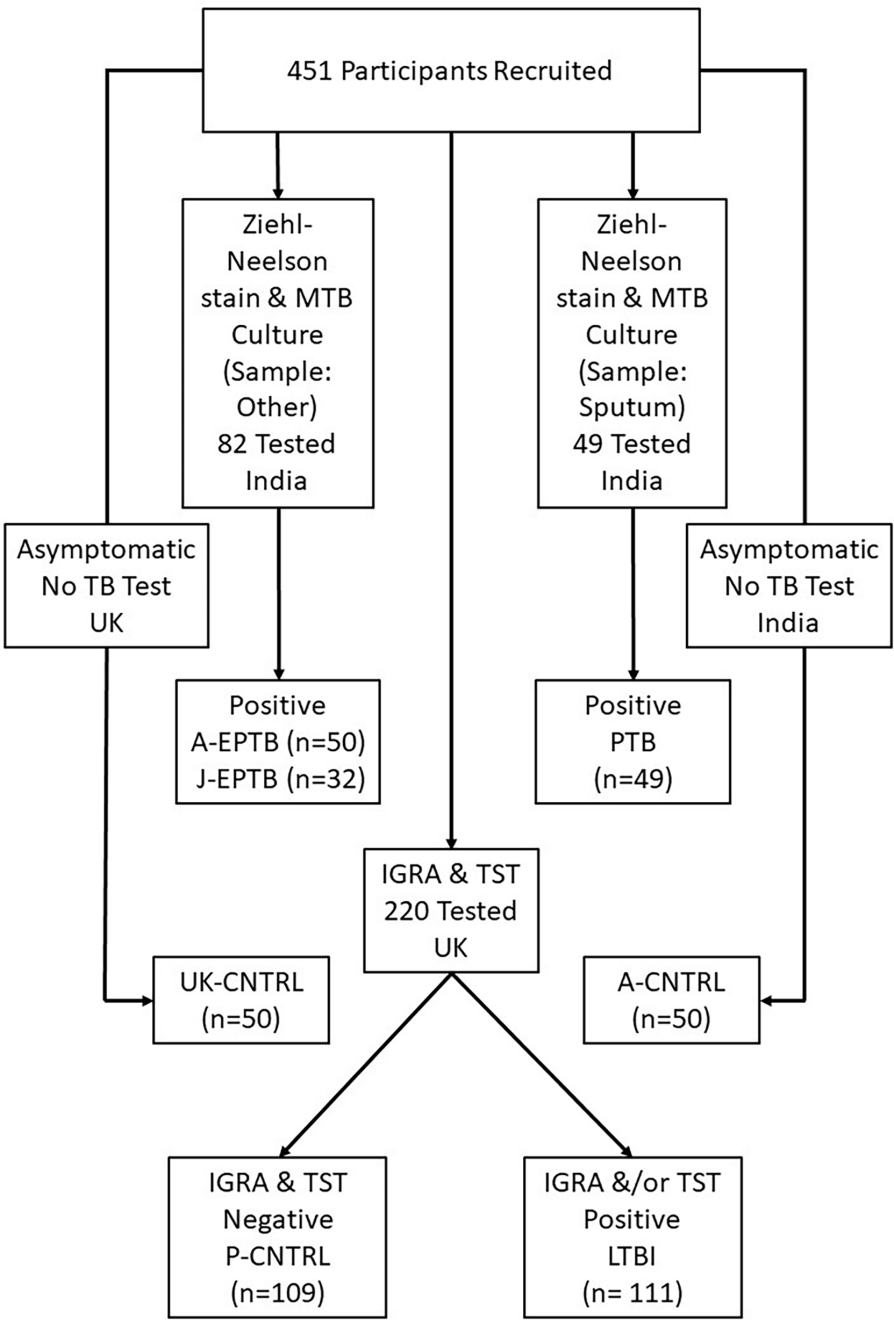
Flowchart of patient and control cohort used in the study.

A total of 452 peripheral blood samples were collected from three cohorts of TB patients – with pulmonary TB (PTB) or extra-pulmonary TB (EPTB) collected at two geographically distant areas in Northern and Southern India, at AIIMS, New Delhi (A-EPTB) or JIPMER, Puducherry (J-EPTB); latent TB (LTBI) and control groups (P-CNTRL), collected as part of the PREDICT TB study and two other control groups collected at the partner site AIIMS in New Delhi (A-CNTRL) or in the UK [UK-CNTRL (First Link Ltd., Wolverhampton, UK)]. All TB patients included in the study from India were > 16 years of age. PTB patients were recruited on the basis of sputum Ziehl Neelsen stain (ZN) positivity for acid-fast bacilli (AFB) and eventual culture positivity for TB. EPTB patients were recruited on the basis of ZN positivity for acid-fast bacilli (AFB) and eventual culture positivity for TB, sampled at a body site other than the lung. A description of recruitment and inclusion criteria of individuals to the PREDICT-TB study has been described previously elsewhere (42, 43). Details of individuals from the PREDICT-TB study in the LTBI group, who progressed to active TB disease have been published recently by Gupta et al. (44). Median time to TB disease among the progressors was 188 days (interquartile range, 76–488 d). Controls collected at all sites were > 16 years of age and recruited on the basis on no outward signs of respiratory disease or other infectious disease conditions (asymptomatic). UK-CNTRLs were less than 55 years of age and certified non-reactive to human immunodeficiency (HIV), Hepatitis B and C by approved antigen or antibody enzyme-linked immunosorbent assay (ELISA) methods. The UK-CNTRL and the A-CNTRL control group were not tested for TB. P-CNTRLs were negative for interferon-γ release assay (IGRA); QuantiFERON^®^ TB Gold In-Tube [(QFT-GIT) QIAGEN GmbH, Hilden, Germany], T-SPOT^®^.TB [(T.SPOT) Oxford Immunotec Ltd, Oxford, UK)] and tuberculin skin test (TST) (43). LTBI samples were identified as being variably positive for all three tests. All patient and control sample details are given in detail in **Supplementary Information S1**, **Table S1.1** and summarised in **Table 1** and study details in **Figure 1**. All ethical approvals for the study were in place prior to sample collection, as described previously (13). Blood samples were collected by venepuncture in lithium heparin tubes and stored at -80°C prior to use.

**Table 1:** Summary of the number of patients per group for all patient and control samples and affiliations with collaborating site in the study.

Sample Group	Description	Region of origin	Number of samples (n)
UK-CNTRL	UK Negative Controls	First Link Ltd., Wolverhampton, UK (low incidence region)	50
P-CNTRL	PREDICT TB Controls	London, UK (PREDICT TB study)	109
LTBI	PREDICT TB Latent TB	London, UK (PREDICT TB Study)	111
A-CNTRL	AIIMS Negative Controls	AIIMS, New Delhi, India (High incidence region)	50
A-EPTB	AIIMS Extra Pulmonary TB	AIIMS, New Delhi, India	50
J-EPTB	JIPMER Extra Pulmonary TB	JIPMER, Puducherry, India	32
PTB	AIIMS Pulmonary TB	AIIMS, New Delhi, India	49

### 2.2 Sample Processing

TB infected blood samples were processed at Containment Level 3 and control samples at Containment Level 2 in laboratories at UKHSA, Porton UK or JIPMER, India. In short, whole blood samples were thawed at room temperature, 2 ml aliquots were then transferred to tubes containing 8ml of Invitrogen Cell Extraction Buffer (Life Technologies, UK). These were mixed by inversion and placed on ice for 30 minutes to lyse. All blood lysates were filtered through Millex GP 0.22 um syringe filters, aliquoted and stored at -80°C prior to further use.

### 2.3 ELISA Assays

Quantification of nine candidate protein biomarkers was conducted using commercially available ELISA assay kits (MyBioSource, USA). The detection range of each assay was as follows; CALCOCO2 (0.78-100ng/ml), CD274 (0.156 -10ng/ml),CD52 (39-5000ng/ml), GBP1 (12.5-1600pg/ml), IFIT3 (0.78-100ng/ml), SAMD9L (1.56-200ng/ml), and TMEM49 (31.2-1000 pg/ml), IFITM3 (3.125-100ng/ml) and SNX10 (6.25-200ng/ml) respectively. All lysed blood samples tested were diluted 1/5 in sample dilution buffer, except for CD52 and GBP1 ELISAs which were tested undiluted. Out of range (high) samples were diluted appropriately in PBS and re-tested. ELISA results were read using a FLUOStar Omega plate reader (BMG Labtech) at UKHSA Porton Down and a FLUOStar Optima plate reader (BMG Labtech) at JIPMER. Raw data were exported using FLUOStar OMEGA MARS analysis software in Excel format, prior to further analysis (given in **Supplementary Information S1**, **Table S1.2**).

### 2.4 Data Analysis and Normalisation

Numeric absorbance values from the standard curve, derived from known concentrations of antigen supplied with each ELISA kit, were used to plot 5-parameter logistic curves, from which protein concentrations were interpolated. These were then corrected for dilution factor, as required. Standard curves run on individual plates were cross compared to determine inter-assay variation. Data outputs between plates were normalised using the y-midpoint intersection value, extrapolated from each plate’s standard curve. In short, the midpoint was calculated using the highest and lowest data points on the y-axis of each standard curve. A value (χ) was calculated by division of the y-midpoint value of the first assay plate standard curve and the y-midpoint values of each subsequent standard curve respectively. Raw test data output values were then corrected by multiplication using the χ value (given in **Supplementary Table File S1: Table S1.2**).

### 2.5 Data Analyses

#### 2.5.1 Statistical Analyses

Transformed data values were further analysed using Microsoft Excel for Microsoft 365 MSO (Excel 365), Sigmaplot version 20 (SP20), GraphPad PRISM version 9.0 (GPP9), the statistics software package ‘R’ x 64 3.4.0 Software (‘R’), or the bioinformatics software package GeneSpring™ 14.9 ((GX14.9) Agilent). Summary statistics analyses and boxplot graphical outputs depicting median, min/max and interquartile ranges were generated using SP20. T-Tests between paired groups (two-tailed with unequal variance) and graphical outputs depicting average values and standard error were conducted using Excel 365. Correlation coefficients were calculated using the correlation matrix from multiple variable analyses function (non-parametric spearman correlation, 2-tailed) using GPP9. Normalised data were imported into GX14.9 with no further modification for multivariate analysis of variance (MANOVA), using Benjamini [Hochberg false discovery rate correction (BH FDR p ≤ 0.05)], T Test (unpaired, unequal variance, no FDR correction) and fold-change analyses.

#### 2.5.2 Random Forest Modelling and ROC Curve Analyses

The performance of each candidate biomarker for discriminating between control and TB disease groups, was determined according to ROC ‘area under the curve’ (AUC) values, calculated using the ‘ROCR’ package function in ‘R’ and the ROC analysis tool of SP20. Cut-off values were predicted by measuring the optimal accuracy of the curve, from which sensitivity and specificity values were calculated. Identification of best performing biomarker panel combinations were predicted by Random Forest modelling using the randomForest package in ‘R’. Models were performed to classify all controls from Active TB (classifying PTB and EPTB as separate groups) and all controls from PTB. Data were split (75% training set, 25% testing set), with samples missing data excluded from the analysis. For biomarker combination selections, variables were ranked on decrease in Gini scores. Composite panel scores were calculated from simple additive algorithms consisting of panels of best performing biomarkers from which composite ROC curve analyses were performed to determine optimal best performing panels of biomarkers. Diagnostic performance of these algorithms was also assessed using Sensitivity, Specificity, Cut-off values and Likelihood ratios.

## 3 Results

### 3.1 Evaluation of Individual Protein Biomarker ELISA Data

Individual protein biomarker expression within each control and TB disease group was analysed using the summary statistics function in SP20 (**Supplementary Information File S1**, **Table S1.3**; UK-CNTRL, **Table S1.4**; P-CNTRL, **Table S1.5**; A-CNTRL, **Table S1.6**; A-EPTB, **Table S1.7**; J-EPTB, **Table S1.8**; LTBI, **Table S1.9**; PTB). The performance of the assays was generally good, although there are some missing replicates from assay failures. Assay Coefficients of Variability (%CVs) were generally low, although they varied with respect to the biomarker targets and groups, which may reflect either innate or disease-associated expression variation between group individuals.

### 3.2 Statistical Analysis of Individual Protein Biomarker Expression Profiles

Median, min max and interquartile range expression values are given in boxplot format in **Figure 1** (with a summary of the numeric values given in **Supplementary Information Table File S1** and **Table S1.10** and mean/standard error graphical depictions given in **Supplementary Information Figure File S1** and **Figures S1.1, S1.2**). Good protein expression was observed for most of the biomarker targets, with increased expression for most of the targets in the active TB groups except for TMEM49 and CD52 (which were generally lover in the TB disease groups compared with controls). The A-CNTRL group exhibited higher expression of most of the protein targets assayed than the other control groups. MANOVA analysis across all biomarker targets and groups determined that all nine biomarkers SAMD9L, CALCOCO2, GBP1, IFITM3, SNX10, IFIT3, CD52, CD274 and TMEM49 in the study were significantly differentially-expressed across the groups (**Table 2**). Fold-change analysis using the A-CNTRL group as baseline, revealed the relative differences in expression between this and the other groups. This group was selected as comparator since this is the most likely group reflecting a baseline level of expression against which patients in regions of high endemic TB would be assessed. This analysis also highlighted differences between this control group and the UK-CNTRL, P-CNTRL and LTBI groups, with lower expression of most biomarkers in these groups compared with the A-CNTRL group, except for TMEM49. Pairwise statistical analysis between groups confirmed these expression differences (**Supplementary Information File S1** and **Table S1.11**), with varied expression observed across groups (**Figure 1** and **Supplementary Information File S1** and **Table S1.11**).

**Table 2:** p-values from MANOVA analysis and fold-change data (using the A-CNTRL group as baseline control) across all biomarkers and groups 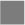 fold-change ≥ 2.5, 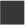 fold-change ≥ 1.5, 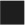 fold-change (white text) ≤ -1.5, ■ fold-change (white text) ≤ -2.5.

Protein Target	Description	MANOVA corrected (BH FDR) p value All Groups	MANOVA uncorrected p value All Groups	PTB vs A-CNTRL Fold Change	J-EPTB vs A-CNTRL Fold Change	A-EPTB vs A-CNTRL Fold Change	LTBI vs A-CNTRL Fold Change	UK CNTRL vs A-CNTRL Fold Change	P-CNTRL vs A-CNTRL Fold Change
**CALCOCO2**	calcium binding and coiled-coil domain 2	0.00E+00	0.00E+00	**3.53**	1.20	1.17	-1.42	-1.38	-1.66
**SAMD9L**	sterile alpha motif domain containing 9 like	0.00E+00	0.00E+00	**3.22**	1.72	1.08	-2.31	-1.36	-2.13
**IFITM3**	interferon induced transmembrane protein 3	0.00E+00	0.00E+00	**2.65**	1.31	1.14	-1.89	-1.47	-1.90
**IFIT3**	interferon induced protein with tetratricopeptide repeats 3	0.00E+00	0.00E+00	1.97	**2.94**	2.19	**-9.85**	**-9.19**	**-16.82**
**GBP1**	guanylate binding protein 1	3.13E-39	2.09E-39	1.92	-1.22	1.20	-1.92	-1.51	-1.90
**SNX10**	sorting nexin 10	4.16E-37	3.23E-37	1.69	1.29	-1.03	-1.59	1.07	-1.68
**CD274**	CD274 molecule	2.81E-42	1.56E-42	1.63	-1.11	-1.01	-1.19	-1.19	-1.19
**TMEM49**	VMP1 vacuole membrane protein 1	1.09E-16	9.68E-17	1.25	1.83	-1.32	1.45	1.97	1.53
**CD52**	CD52 molecule	3.09E-02	3.09E-02	-1.49	-1.24	-1.56	-1.64	-1.52	-1.50

Significant up-regulation of most biomarkers was observed for the active disease groups, when compared with the UK-CNTRL and P-CNTRL groups, but this was somewhat reduced in comparison with the A-CNTRL group. High average expression of SAMD9L, CALCOCO2, GBP1, IFITM3 and SNX10 was observed in the PTB group compared to the EPTB and other groups, in contrast IFIT3 expression was higher in the EPTB groups. There were significant differences in expression between the two EPTB groups, for SAMD9L, GBP1, SNX10 and TMEM49 expression. Average CD52 expression was higher in the UK-CNTRL and A-CNTRL controls compared with active disease groups. Overall protein expression was significantly lower in the LTBI than all control and Active TB groups for all targets. LTBI as a combined group exhibited significant differences from the UK-CNTRL control group for SAMD9L, GBP1, IFITM3 and SNX10, for IFIT3 with its equivalent control group P-CNTRL and all biomarkers with the A-CNTRL group (**Supplementary Information File S1** and **Table S1.11**).

During study follow up, several individuals (8/106) from the LTBI group were found to have progressed to active disease (LTBI_PR), consistent with the 5-10% lifetime risk of reactivation of TB for an individual with documented LTBI, with the majority developing TB disease within the first five years after initial infection (45). These were separated from LTBI non-progressors (LTBI_NPR) and analysed as a discrete group. T tests revealed expression differences between the LTBI_PR group, the LTBI_NPR and matched P-CNTRL groups for IFITM3, IFIT3 and CD52 (**Supplementary Information File S1** and **Table S1.12**), particularly for CD52 which was approximately two-fold lower in expression in the LTBI_PR group compared with both the LTBI_NPR and P-CNTRL groups (**Supplementary Information File S2** and **Figure S2.2**). A near significant expression difference was observed for CD274 between the P-CNTRL and the LTBI_PR groups.

### 3.3 Correlation of Biomarker Expression With QuantiFERON, T-SPOT.TB and Tuberculin Skin Test in PREDICT TB Study Samples

Normalised protein target expression data were imported without further modification into the bioinformatics software GX14.9. The P-CNTRL and LTBI groups were annotated with regard to their QFT-GIT, T.SPOT and TST status. Individual T Test analyses (uncorrected, no FDR) were conducted between the two groups for differential biomarker target expression correlating with QFT-GIT, T.SPOT or TST status (significance p values are given in **Supplementary Information File S1** and **Table S1.13**). MANOVA analyses could not be conducted between LTBI_PR and LTBI_NPR groups due to significant variations in group size. QFT-GIT positivity correlated with GBP1 (p-value 0.0196) and TMEM49 (p-value 0.0356) expression and to a lesser degree of significance with IFIT3 (p-value 0.0544) and SNX10 (p-value 0.079). T.SPOT positivity showed a weak correlation with IFIT3 (p-value 0.095) and TST with CALCOCO2 (p-value 0.0754).

### 3.4 Correlation Matrix Analysis of Protein Biomarker Inter-Relationships

**Figure 2:**
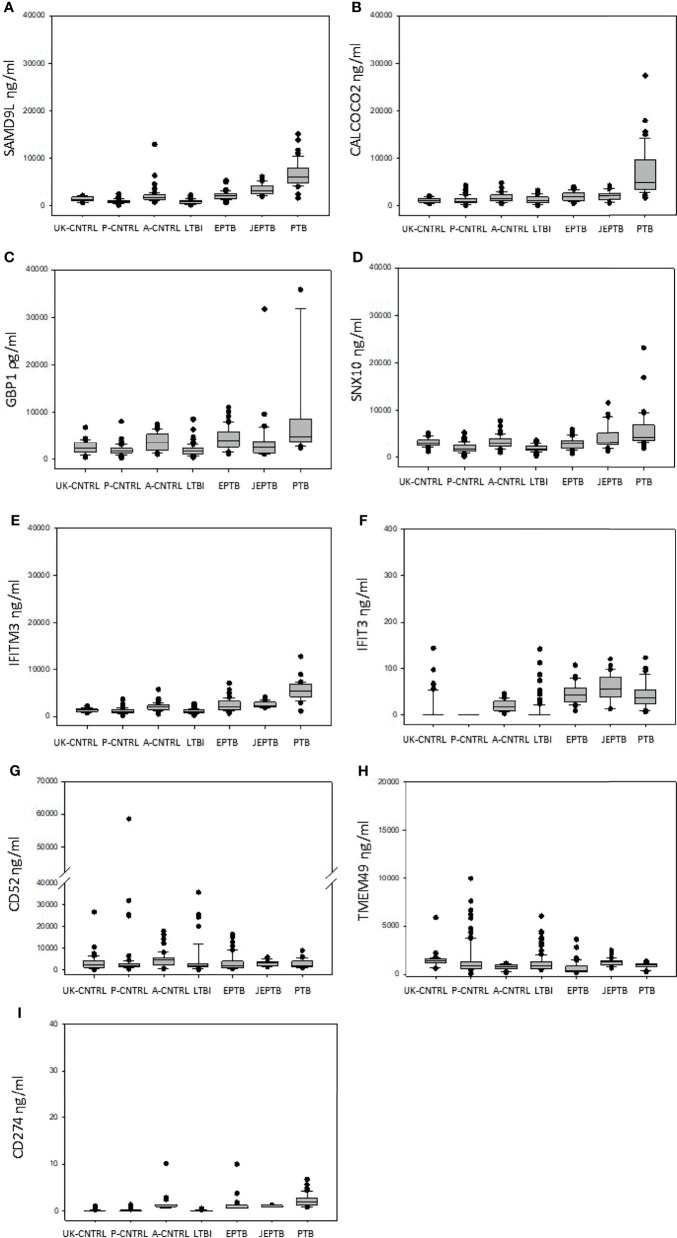
Graphical boxplot depiction of biomarker expression across all control and patient groups (displaying median, minimum, maximum and interquartile expression range) **(A)** SAMD9L **(B)** CALCOCO2 **(C)** GBP1 **(D)** SNX10 **(E)** IFITM3 **(F)** IFIT3 **(G)** CD52 **(H)** TMEM49 **(I)** CD274.

Correlation coefficients were calculated using the ‘correlation matrix from multiple variable analyses function’ (non-parametric 2 tailed, Spearman correlation) using GPP9. These are given in heatmap image format in **Figure 2** and corresponding correlation and significance p values in **Supplementary Information File S1** and **Table S1.14**. These showed complex patterns of interactions, varying across the control and disease groups. However, some consistent patterns were observed, with strong correlations between IFITM3 and SAMD9L, CALCOCO2 and SNX10 in all control groups, with reduced positive interaction between IFITM3 and SNX10 in the LTBI, J-EPTB and PTB groups. IFITM3 positive interactions with SAMD9L, CALCOCO2, GBP1 and SNX10 were very reduced in the PTB group. There were also increasingly negative correlations between GBP1 and SNX10, IFIT3 and TMEM49 in the A-EPTB, J-EPTB and PTB groups, GBP1 and CD274 in the E-EPTB group and GBP1 and CD52 in the J-EPTB and PTB groups. Pronounced correlation expression differences were observed between the LTBI_PR and LTBI_NPR groups, particularly for CD52, which exhibited a strong negative correlation with all other biomarkers except CD274 and itself in the LTBI_PR group. These relationships may correlate with shifting immune profiles in the transition from control to latent and then active TB disease states.

### 3.5 Accuracy of Individual Biomarkers From Whole Blood for Detection of TB Infection

Paired ROC curve analyses were conducted between groups for individual protein biomarkers to determine the relative accuracy of each candidate for discrimination across infected and uninfected groups (given in full in **Supplementary Information File S2**, **Table S2.2** and summarised in **Table 3**). As well as comparisons between individual groups, comparisons were made between all control groups combined (UK-CNTRL/P-CNTRL/A-CNTRL), all EPTB groups and all Active TB groups (A-EPTB/J-EPTB/PTB). Seven of the nine biomarkers were able to discriminate all Active TB from all control groups with good accuracy (ROC ≥ 0.7). IFIT3, IFITM3 and SAMD9L achieved excellent discrimination for Active TB when compared to individual control groups with AUC values between 0.85-1. CD274 performed well for discriminating Active TB from UK-CNTRL and P-CNTRL but fared less well for Active TB from A-CNTRLs. High number of positives were observed in the A-CNTRL compared with the UK-CNTRL and P-CNTRL groups for most biomarkers, suggesting that this group may be heterogeneous and may contain infected or exposed individuals.

**Table 3:** Summary of ROC curve values for biomarker expression for all control and TB disease group combinations 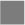 ROC curve value ≥ 0.9, 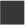 ROC curve value ≥ 0.8, 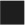 ROC curve value ≥ 0.7, ■ ROC curve value (white text) ≤ 0.3.

GROUP COMPARISON	CALCOCO2	CD274	CD52	GBP1	IFIT3	IFITM3	SAMD9L	SNX10	TMEM49
UK-CNTRL vs Active TB	0.859	**0.988**	0.520	0.727	**0.907**	0.889	**0.907**	0.611	0.206
UK-CNTRL vs A-EPTB	0.762	**0.978**	0.477	0.764	**0.906**	0.770	0.860	0.444	0.137
UK-CNTRL vs All EPTB	0.781	**0.982**	0.520	0.727	**0.911**	0.850	0.787	0.500	0.241
UK-CNTRL vs J-EPTB	0.809	**0.987**	0.590	0.618	**0.921**	**0.976**	**0.974**	0.590	0.404
UK-CNTRL vs LTBI	0.519	0.534	0.437	0.368	0.492	0.271	0.190	0.138	0.272
UK-CNTRL vs PTB	**0.987**	**0.997**	0.497	**0.900**	**0.901**	**0.953**	**0.987**	0.798	0.148
P-CNTRL vs Active TB	0.853	**0.990**	0.561	0.826	**1.000**	**0.932**	**0.970**	0.838	0.436
P-CNTRL vs A-EPTB	0.767	**0.987**	0.489	0.861	**1.000**	0.870	**0.928**	0.743	0.248
P-CNTRL vs All EPTB	0.779	**0.986**	0.561	0.826	**1.000**	**0.906**	**0.955**	0.780	0.412
P-CNTRL vs J-EPTB	0.797	**0.985**	0.678	0.725	**1.000**	**0.962**	**0.997**	0.841	0.669
P-CNTRL vs LTBI	0.567	0.515	0.452	0.496	0.585	0.504	0.454	0.513	0.484
P-CNTRL vs PTB	**0.976**	**0.997**	0.535	**0.968**	**1.000**	**0.976**	**0.997**	**0.937**	0.476
A-CNTRL vs Active TB	0.736	0.489	0.339	0.537	0.817	0.737	0.793	0.626	0.585
A-CNTRL vs A-EPTB	0.587	0.674	0.337	0.537	0.810	0.568	0.593	0.477	0.363
A-CNTRL vs All EPTB	0.587	0.681	0.339	0.580	0.843	0.622	0.705	0.527	0.554
A-CNTRL vs J-EPTB	0.637	0.690	0.342	0.411	0.897	0.707	0.879	0.608	0.851
A-CNTRL vs LTBI	0.360	**1.000**	0.292	0.224	0.103	0.127	0.087	0.191	0.600
A-CNTRL vs PTB	**0.950**	0.178	0.321	0.727	0.775	**0.930**	**0.941**	0.793	0.637
ALL CNTRLS vs Active TB	0.826	0.875	0.497	0.733	**0.935**	0.874	**0.913**	0.733	0.421
ALL CNTRLS vs A-EPTB	0.723	0.826	0.448	0.770	**0.933**	0.774	0.813	0.608	0.251
ALL CNTRLS vs All EPTB	0.738	0.827	0.497	0.733	**0.942**	0.822	0.872	0.653	0.408
ALL CNTRLS vs J-EPTB	0.762	0.827	0.575	0.624	**0.957**	0.897	**0.963**	0.725	0.654
ALL CNTRLS vs LTBI	0.506	0.396	0.409	0.400	0.449	0.368	0.302	0.346	0.465
ALL CNTRLS vs PTB	**0.972**	**0.955**	0.473	0.893	**0.923**	**0.960**	**0.981**	0.869	0.442
LTBI vs ACTIVE TB	0.711	**1.000**	0.583	0.850	**0.932**	**0.937**	**0.934**	0.872	0.448
LTBI vs A-EPTB	0.721	**1.000**	0.523	670.67	**0.929**	0.875	**0.996**	0.770	0.251
LTBI VS J-EPTB	**0.974**	**1.000**	0.690	**0.951**	**0.945**	**0.976**	**0.998**	0.880	0.693
LTBI vs PTB	0.812	**1.000**	0.573	0.845	**0.926**	**0.976**	**0.973**	**0.972**	0.490

Predicted cut-off values were selected to achieve the best sensitivity and specificity of the individual biomarkers for discriminating infected from non-infected individuals. SAMD9L was observed to be the best performing biomarker for PTB [ROC AUC values; PTB vs UK-CNTRL (0.987), P-CNTRL group (0.997), A-CNTRL (0.911), and combined control groups (0.981)]. IFIT3 consistently achieved the highest AUC values for discrimination of EPTB from control groups (ROC AUC values; EPTB vs UK-CNTRL (0.911), P-CNTRL group (1.00) A-CNTRL (0.843) and combined control groups (0.942). Only CD274 showed any reasonable performance for the LTBI group using the A-CNTRL group as comparator (1.00). There appears to be an inverse correlation with CD52, GBP1, IFIT3, IFITM3, SAMD9L, SNX10 and TMEM49 with the other individual control groups for LTBI.

### 3.6 Improved Performance Combination Biomarker Panels

Single biomarkers were observed to show good performance across all individual groups. However due to the heterogeneity of expression across all TB disease presentations, combined panels using best performing biomarkers were investigated to increase diagnostic performance for all forms of Active TB (including EPTB) and PTB. CALCOCO2, GBP1, IFIT3, IFITM3, SAMD9L and SNX10 were selected for further combinatorial analysis. CD52 and TMEM49 were excluded due to their poor individual ROC curve performances. CD274 was also excluded, as despite its good ROC curve performance for some presentations, its fold-change expression range was very low.

Random forest modelling was performed using the randomForest ‘R’ package to classify both control and Active TB groups and control and PTB only. Data were randomly split for analysis (75% training and 25% testing), with samples missing data excluded from the analysis. For classification of Controls, EPTB and PTB, the randomForest model showed an Out-Of-Bag (OOB) estimate of error rate of 10.98% with 3 variables tried at each split. SAMD9L, IFITM3 and IFIT3 were ranked highest in importance for the classification of these groups individually [**Figure 4A** (I); **Supplementary Information S2 Table S2.1** (I)]. For classification of PTB from controls only, the randomForest showed an OOB estimate of error 3.11% with SAMD9L, IFITM3 and CALCOCO2 revealed as variables of most importance with 3 variables tried at each split [**Figure 4A** (II); **Supplementary Information S2 Table S2.1** (II)].

Composite panel scores were calculated using simple additive algorithms consisting of the predicted top performing biomarkers to determine in which combination they best discriminated (1) all active TB groups from all control groups and (b) PTB from all control groups (**Supplementary Information File S2** and **Table S2.3**).

One panel combination using a simple additive algorithm of all 6 biomarkers showed superior performance for discrimination of all controls from all active TB;


(300×IFIT3)×(3×SAMD9L)+GBP1+IFITM3+SNX10+CALCOCO2

**Figure 5** shows composite box and dotplots of (A) all combined controls vs all combined active TB, (B) all individual control groups vs all individual active TB groups. ROC curve analyses demonstrated the high performance of this combination for PTB (ROC = 0.9885) compared with the A-EPTB and J-EPTB groups [**Figure 4B** (I)]. Reduced performance was observed for all combined controls vs all combined active TB (ROC = 0.9552).

**Figure 3:**
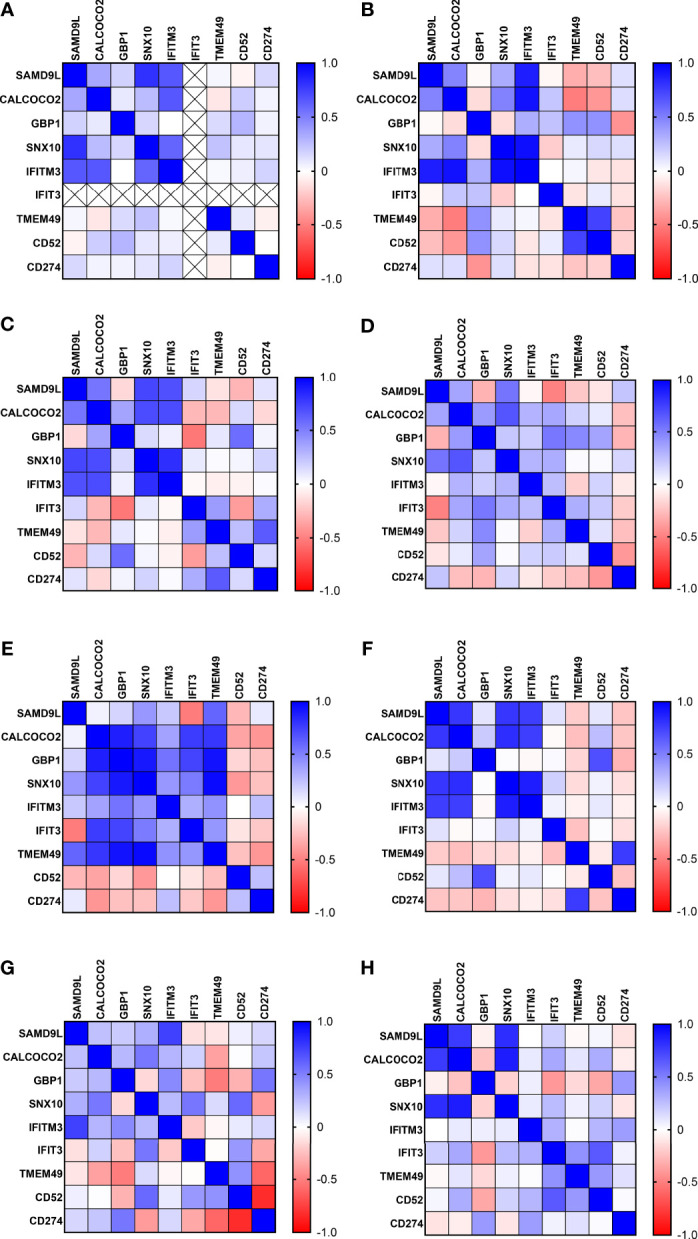
Correlation matrix analysis of protein biomarker inter-relationships in all control and TB disease groups **(A)** UK-CNTRL **(B)** P-CNTRL **(C)** A-CNTRL **(D)** LTBI non-progressors **(E)** LTBI progressors **(F)** J-EPTB **(G)** A-EPTB **(H)** PTB.

**Figure 4:**
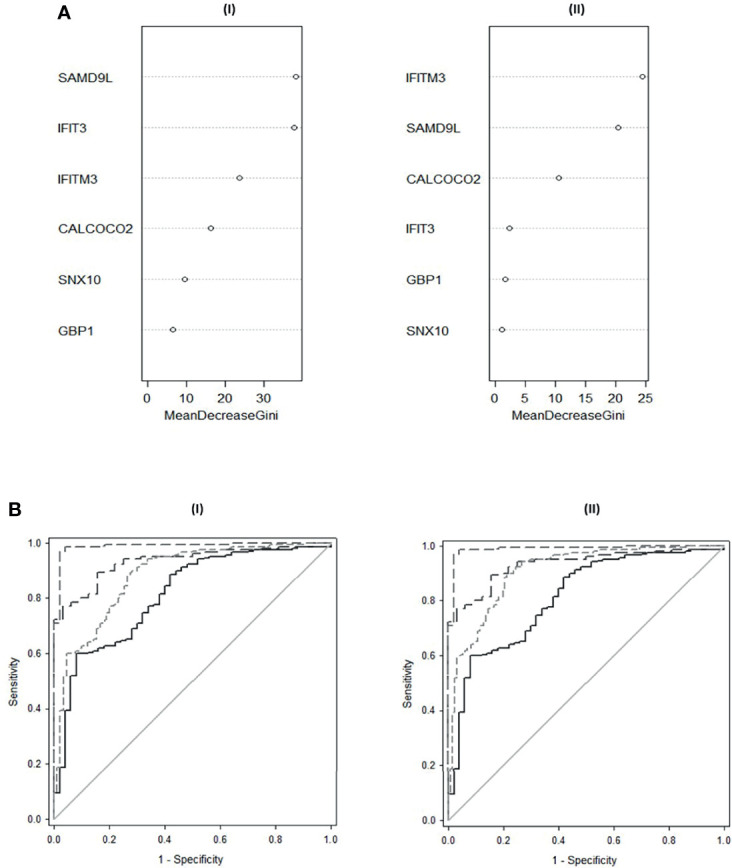
**(A)** Variable Importance Plot of decrease in Gini scores as measured by Random Forest for **(I)** classification of All Controls, EPTB and PTB **(II)** Classification of All Controls and PTB**. (B)** ROC curves of composite panel scores generated from **(I)** the complex 6-plex panel for discrimination of individual TB groups and combined Active TB from All controls **(II)** the simple 3-plex panel for the discrimination of individual TB groups and combined Active TB from All controls. All controls vs A-EPTB ───, all controls vs J-EPTB ─ ─, all controls vs PTB ─ ─ ─, all controls vs all combined active TB ─ ─ ─ ─.

**Figure 5:**
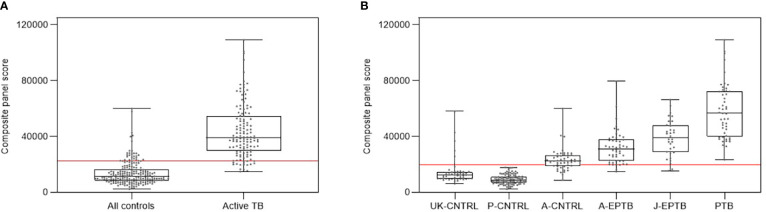
**(A)** Combined box and scatter plot graphical depictions of composite panel score of the complex 6-plex biomarker panel between all controls and all active TB groups combined, displaying the cut-off value y=22361 for discrimination of all active TB groups from all controls with 90.1% sensitivity and 85.7% specificity **(B)** Combined box and scatter plot graphical depictions of expression of the complex 6-plex biomarker panel between individual control and active TB groups displaying the cut-off value y=19698 for discrimination of all active TB groups from all controls with 95.4% sensitivity and 81.3% specificity.

A refined, simplified panel of these six markers also exemplified all controls from PTB;


CALCOCO2+SAMD9L+IFITM3

**Figure 6:**
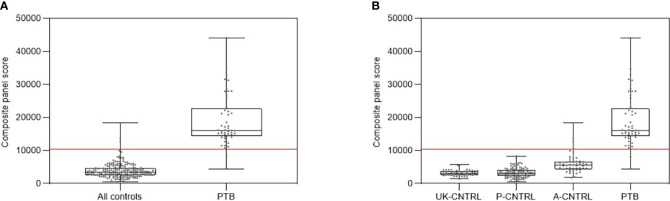
**(A)** Combined box and scatter plot graphical depictions of composite panel score of the simple 3-plex biomarker panel between all controls and PTB, displaying the cut-off value y= 10389 for discrimination of PTB from all controls with 95.9% sensitivity and 98.6% specificity. **(B)** Combined box and scatter plot graphical depictions of expression of the simple 3-plex biomarker panel between individual control and PTB displaying the cut-off value y= 10389 for discrimination of PTB from all controls with 95.9% sensitivity and 98.6% specificity.

**Figure 6** shows composite box and dotplots of (A) all combined control groups vs PTB, (B) all individual control groups vs PTB. ROC curve analysis demonstrated the high performance of this combination for PTB (ROC = 0.9894) compared with the A-EPTB and J-EPTB groups (**Figure 4B** (II)). Reduced performance was also observed for all combined controls vs all combined active TB (ROC = 0.9079).

Calculated sensitivity, specificity and positive and negative predictive values for the panels were compared with the minimum and optimum technology product profiles for the TB triage test (minimum; 90% Sensitivity/70% Specificity, optimum; 95% Sensitivity/80% Specificity), given in **Supplementary Information File S2** and **Table S2.4** and the TB confirmatory test (minimum; 65% Sensitivity/98% Specificity, optimum; (i) sputum positive PTB 98% Sensitivity/98% Specificity (ii) EPTB 80% Sensitivity/98% Specificity), given in **Supplementary Information File S2** and **Table S2.5**. These results demonstrated that the panels meet the minimum performance criteria for the combined controls vs all active TB groups, with variation in performance observed across the different individual control groups and subtypes of disease. The optimum performance criteria for the triage test was achieved using both panels for many of these latter pairwise comparisons, but the optimum performance criteria was only met for EPTB group comparisons (because of the reduced sensitivity performance level (80%) for EPTB). The minimum requirements were met for the confirmatory test for most of the pairwise comparisons, but the optimum requirements only for a few using the P-CNTRL or UK-CNTRL groups as comparators e.g. P-CNTRL or UK-CNTRL vs PTB for the simplified panel.

## 4 Discussion

Here we describe a pilot study to assay select, previously-validated, TB-associated immune mRNA biomarkers CALCOCO2, CD274, CD52, GBP1, IFIT3, IFITM3, SAMD9L, SNX10 and TMEM49 from our previous study (13), as protein targets. These were assayed using commercial ELISA assays at UKHSA, Porton UK and JIPMER, Puducherry, India, using whole lysed blood samples from individuals with suspected LTBI or ATB infection and three groups of controls. Most of the proteins showed expression in the ηg/ml range except GBP1 and TMEM49 which were in the ρg/ml range. Mean and median analyses of the protein biomarkers showed increasing, incremental expression patterns from the P-CNTRL, UK-CNTRL and the A-CNTRL groups through to the EPTB and PTB groups. Higher than expected biomarker expression was also seen in some individuals in the control groups. The A-CNTRL group showed greater number of individuals expressing higher levels of these protein biomarker targets than the other two control groups. Expression in the P-CNTRL and LTBI groups were low for most of the biomarkers and they were highly similar to one another. Few differences were seen between these two groups except with expression of IFIT3, for which there was no recorded expression in the P-CNTRL group, in the dynamic range of the assay. IFIT3 expression in the combined LTBI group exhibited weak statistical correlation with T.SPOT and QFT-GIT but not TST positivity. GBP1 and TMEM49 exhibited significant correlation with QFT-GIT positivity and to a lesser extent SNX10. Expression of IFIT3 appears to correlate with the LTBI_NPR group and not the LTBI_PR group. CALCOCO2 appeared to show weak correlation with TST. These may reflect diversity in the functionality between the assays and this is reflected in the positivity profiles within the LTBI group (**Supplementary Information S1** and **Table S1.1**). Abubakar et al. noted that combination strategies including all three tests were significantly superior predictors of progression in LTBI patients (43), implying that the use of individual tests gave mixed results. They concluded that multi-testing strategies showed superior performance. The results presented here may imply that the three different tests may demonstrate immunological bias.

Expression levels of SAMD9L CALCOCO2, GBP1, SNX10, IFITM3 and IFIT3 were good and correlated well with TB disease. TMEM49 and CD274 showed more modest expression and appeared more variable in their expression profiles. IFIT3 expression levels were generally low but showed a high degree of specificity to TB disease groups and also some expression in individuals in the A-CNTRL group. As these control samples were collected from a high incidence region of TB infection where the carriage rate of infection is expected to be ≥ 40%, it is postulated that this group could be a heterogeneous population containing a proportion of previously-unidentified TB-exposed or infected individuals. This may highlight issues in discrimination of uninfected and infected individuals from high TB burden regions. However, individuals with increased biomarker abundance above calculated thresholds would be likely candidates for follow-up for suspected TB infection, even if asymptomatic.

Expression differences between the groups were confirmed using MANOVA and pairwise T-tests for nearly all of the biomarkers except CD52, which showed differences between the A-CNTRL, J-EPTB and PTB groups only. Overall, SAMD9L, CALCOCO2, GBP1, IFITM3, IFIT3 and SNX10 showed the most significant expression increase in all the ATB compared to all control groups, with very high expression in the PTB group. Expression differences were observed between the two EPTB groups for SAMD9L, GBP1, SNX10 and TMEM49, suggesting regional variations between these groups. The A-EPTB group showed higher expression of CALCOCO2, GBP1 and IFITM3 than the J-EPTB group, which in contrast exhibited higher expression of SAMD9L, SNX10, IFIT3 and TMEM49. CD274 expression was similar in both groups. CALCOCO2, IFITM3 and CD274 show the most significant expression differences between the PTB group and the two EPTB groups. IFIT3 is elevated in all three disease groups, but is slightly higher in the EPTB groups. These former three biomarkers may be correlated with disease progression and/or severity and could be used to discriminate between these two subtypes of disease, in conjunction with IFIT3 which is expressed in all subtypes of TB. We were able to distinguish LTBI from active TB and high Incidence region controls using most biomarkers but were unable to discriminate LTBI from the matched P-CNTRL control group. Differences in expression for CD52 were seen between the LTBI non-progressors and progressors, this biomarker was significantly downregulated in the latter. These results showed promised for further analysis to determine best performing characteristics or diagnostic purposes.

Correlation coefficient analyses showed somewhat complex patterns of interactions; however, several features were noteworthy. There were strong positive correlations between SAMD9L, CALCOCO2, SNX10 and IFITM3 in all the control groups. These interactions appear lessened in the LTBI and J-EPTB groups and the interaction between IFITM3 and these biomarkers s is severely reduced in PTB. GBP1 and TMEM49 show increasing negative interactions in all three disease groups, A-EPTB, J-EPTB and PTB. There was also a strong negative interaction between GBP1 and CD52 in the J-EPTB and PTB groups. This implies that increasing expression of GBP1 may correlate with disease progression and may be negatively influencing CD52 and TMEM49 expression which appears to be interconnected. CD52 expression was down two-fold in the LTBI_PR group and this appeared to have a negative effect on all other biomarkers except CD274. This implies functional impairment or loss of T-cells in the periphery at an early stage of incipient TB progression by mechanisms unknown, but which could include trafficking of antigen-specific T-cells to an emerging site of active infection, inhibition or networked cell death (46, 47). All of these proteins are involved in macro-autophagy, particle uptake, trafficking and digestion through phago-lysosomal pathways and are involved in alternate killing of intracellular pathogens.

SAMD9L is a myeloid tumour suppressor gene, which has been shown to involved in the innate response to viral infection (48) and also plays a role in regulating the response to type I interferons in haematopoietic cells and other cell types e.g. T-cells (49). Studies in mice have shown it controls endocytosis of receptors, homotypic fusion of endosomes, and lysosomal activation (50). Other studies have shown SAMD9L to be upregulated in active TB as compared to LTBI infection (51), it may therefore play a role in intracellular uptake and trafficking of TB bacilli. CALCOCO2 plays a similar role in that it functions as a receptor for ubiquitin-coated bacteria and plays an important role in innate immunity by mediating macro-autophagy (52–54). This suggests a role in targeting TB bacilli for degradation by the innate immune system (54–56), which TB has evolved strategies to evade (57, 58). However, increased CALCOCO2 expression may be a double-edged sword as it also targets the signalling adaptor MAVS for ubiquitination and autophagic degradation, hence inhibiting DDX58-mediated type I interferon signalling through a negative feedback loop (59). SNX10 is also involved in intracellular trafficking and may play a role in regulating endosome homeostasis (60, 61). Therefore, these proteins are intimately inter-related functionally and their dysregulation may have consequences for intracellular bacterial trafficking, bactericidal killing mechanisms and have subsequent deleterious downstream effects on interferon-driven adaptive immune responses.

GBP1, IFIT3 and IFITM3 are also interferon-regulated genes and their upregulation in TB disease groups is significant. Both IFIT3 and GBP1 have been implemented in other previously-published TB diagnostic panels (13). Guanylate binding proteins (GBP) like GBP1 are a large family of IFN-induced hydrolases which are necessary for mediation of host innate immune responses (62, 63). GBP1 functions to elicit divergent host cell death programs in response to infection with intracellular pathogens (64), and increases access to PAMPs. IFIT3 functions to inhibit the function of TLR3 (65), which is involved in recognition of cytoplasmic PAMPS like ‘foreign’ double stranded RNA (66). IFITM3 is a key mediator of the early innate cellular response, however it functions to inhibit phagocytosis, which is beneficial in viral infections, but not with intracellular bacterial pathogens like Listeria which have evolved strategies to exploit its function to avoid phagocyte killing (67). Therefore, upregulation of IFITM3 may inhibit bacterial uptake, but upregulation of GBP1 and parallel downregulation of IFIT3, may lead to GBP-mediated bacterial killing mechanisms and an increase in TLR3-directed immune responses. It is interesting to note the impact of GBP1 on TMEM49 and CD52 and IFITM3 on SAMD9L, CALCOCO2 and SNX10 protein inter-relationships in the PTB group.

SAMD9L, CALCOCO2, SNX10, GBP1, IFIT3, IFITM3 and were all significantly expressed in the active disease groups, making them good candidates for formulation of panels for TB diagnosis. Random Forest modelling and empirical evaluation of many different combinations led to selection of a complex panel, used in combination with the algorithm (300 × *IFIT*3) × (3 × *SAMD*9*L*) + *GBP*1 + *IFITM*3 + *SNX*10 + *CALCOCO*2), which showed good performance for discrimination of all TB disease groups from all controls (ROC = 0.9552, % sensitivity = 90.43, % specificity = 83.97). This was further evaluated against individual disease groups and demonstrated superior performance for discrimination of the PTB disease group (ROC = 0.9885, % sensitivity = 95.22, % specificity = 97.96, at a cut-off value of 28032) and the J-EPTB group from all controls (ROC = 0.9522, % sensitivity = 95.22, % specificity = 81.25, at a cut-off value of 28032). The panel also met the optimum TPP requirements for all controls vs the combined EPTB groups and the A-EPTB and J-EPTB groups individually, at the reduced requirement for the confirmatory test (80% Sensitivity and 98% Specificity) and all controls vs all combined TB disease and vs the PTB group for the minimum requirements for the confirmatory test (65% Sensitivity and 98% Specificity). The likely platform for this panel is a laboratory-based device, due to its larger, more complex biomarker configuration, however there are groups working on multi-analyte LFD devices which may enable future configuration of this larger panel in LFD format (68–70). It would be useful for diagnosis of smear-positive PTB under the minimum TPP requirements (≥ 65% sensitivity) but not optimum requirements (≥ 98% sensitivity), but for diagnosis of smear-negative PTB (optimal requirements ≥ 68% sensitivity) and EPTB (optimal requirements ≥ 80% sensitivity) at the optimum requirements. We were unable to distinguish a panel to identify LTBI from appropriate demographically-matched control groups.

Ideally a biomarker test should be easy to perform and interpret in a health care setting on point of care devices e.g. lateral flow devices. Evaluation of a reduced, simplified panel (*CALCOCO*2 + *SAMD*9*L* + *IFITM*3) more suitable for configuration in this current format was conducted. This showed good performance for discrimination of all TB disease groups from all controls (ROC = 0.9079, %sensitivity = 90.43, %specificity = 76.34), but individually only for the PTB (ROC = 95.22% sensitivity = 95.22, % specificity = 97.96, at a cut-off value of 7574 or ROC = 0.9894% sensitivity = 98.56, % specificity = 95.92, at a cut-off value of 10389) and J-EPTB groups (ROC = 0.933, % sensitivity = 90.43, % specificity = 78.13, at a cut-off value of 6201). It therefore demonstrated useful performance for discrimination of the PTB disease group at both the minimum and optimal WHO triage TPP requirements, for the J-EPTB group at the minimum WHO triage TPP requirement only and the minimum confirmatory test requirement for both. Cut-off thresholds could be adjusted to bias/maximise either the sensitivity or the specificity of the test for PTB diagnosis. This minimum panel shows potential, mainly as a triage test for PTB, but may additionally pick up high CALCOCO2, SAMD9L and IFITM3 expressing EPTB.

Other groups have conducted a recent systematic review of TB Biomarkers and multiple biomarker signatures: MacLean et al. evaluated the quality of biomarker studies and identified most promising biomarkers of active TB for development of POC tests (71). Eleven host protein, blood-based studies were found to meet the WHO’s TPP criteria. However, it was observed that most studies were of insufficient size or did not include a clinically relevant control population, resulting in over-inflated diagnostic performances. Competing blood-based biomarker signatures with potential for development often consisted of multiplex panels of more than 6 biomarkers, currently suboptimal for development of low cost POC triage tests. Jacobs et al. published a 6-host biomarker signature able to diagnose TB disease with a sensitivity of 100% and specificity of 89.3% (34). This 6-host biomarker signature included acute phase proteins CRP, ferritin and PCT typically associated with general inflammation. Although promising, the authors recognised the limitations of the size of the study. A larger cohort of patients was later published with a 7-host biomarker blood-based signature (72), however this study did not include extra-pulmonary TB patients. A similar study has recently been published by Garay-Baquero et al. (30), who identified a 5-panel plasma protein biomarker panel, which could distinguish TB from healthy controls (AUC = 0.93) and other respiratory diseases with a good degree of accuracy (AUC = 0.81).

In the study by Garay-Baquero et al. they state that *‘Current limitations to mainstream serum or plasma proteomics pipelines partly stem from the predominance in protein mass (>95%) of the top 20 most abundant proteins. These high-abundance proteins either mask the presence of or are noncovalently bound to lower abundance proteins with potential clinical relevance. In an effort to overcome this limitation, an initial serum/plasma depletion step to remove such high-abundance proteins is typically employed before the mass spectrometry–based analysis. This plasma proteome analysis strategy has been used in samples from patients with TB. However, this approach will result in the inadvertent loss of a wide spectrum of physiologically important proteins, including those typically encountered in lipid microvesicles, such as exosomes, proteases and their cleavage products, and native peptides such as hormones.* These workers adopted a multidimensional or orthogonal liquid chromatographic separation combined with high-definition mass spectrometry analysis, to enable discovery of low–molecular weight sub-proteome protein targets.

This and many previously published protein target studies have used plasma or serum as sample type. This analytical approach is unlikely to detect peripheral blood, cell-bound targets, unless from lysed cells or if found free in soluble form. Many proteins are likely to be removed during the centrifugation procedure used to generate plasma and serum including cell-associated or cell-bound targets. In this study the sample type used was whole lysed blood. The results presented here may reflect an alternate cell-associated proteome, which is not easily assayed using other methods e.g. mass spectrometry. Cross-comparison of our results with other studies is likely therefore to be discordant, due to these differences in sample type and preparation and analytical methodology.

However, the targets identified in the Garay-Baquero study after validation using ELISA or Luminex assays are observed to exhibit expression levels in a similar concentration range to the protein targets presented in this study (high ρg to ηg/ml concentration range). There may be therefore synergies between different experimental approaches which could be optimised to facilitate future TB diagnostic assay development, particularly for development of a confirmatory test which meets all WHO optimum performance criteria for sputum positive TB (whose stringent criteria no panel has met so far). Further work is required to assess the reproducibility of the protein targets in this study prior to ongoing assay development, however the blood sampling and lysis technology is likely to fit well with POC or LFD-type technologies and assist with assay turnaround time and ease of use.

In summary, we have evaluated 9 biomarkers identified in our previous qPCR study for protein expression in whole lysed blood of TB patients and controls. Our 6-marker panel is showing promise for use for diagnosis of all forms of ATB in a laboratory setting e.g. on based multiplex immunoassays such as Luminex, Fireplex or ELISA or potentially future multianalyte LFDs. Utility of a simplified protein biomarker panel for use on POC devices e.g. current lower-complexity LFDs, showed limited potential for PTB and high expression EPTB patients only. No biomarker panel was identified which showed usefulness for discrimination of LTBI from demographically appropriately matched controls, although a significant difference in CD52 expression was observed in LTBI progressor. However this is unlikely to be useful in regions with a high background of endemic HIV. Further work is required to develop these panels further on suitable platforms and devices.

## 6 Study Limitations

A major limitation of this study was the logistics relating to ELISA assay procurement, shipping and experimental evaluation in two different laboratory settings, with different ambient environments and equipment, leading to some assay replicate failures. Future studies should also include samples from confirmed non-TB mycobacterial infectious or inflammatory diseases such as pneumonia due to other bacterial infectious agents e.g. group A Streptococci, sarcoidosis and other similar systemic inflammatory disorders as well as uninfected groups, to ascertain the specificity of the diagnostic panels for TB. The number of LTBI individuals progressing to active disease was also relatively small and this limited the power of the analysis, as they could not be analysed as a separate group with some of the statistical methods used. Limited demographic information was available for the control volunteers and patients included in the study including age and sex, for the patients HIV, Hepatitis B and C and CMV status and for the control groups incomplete verification of TB, HIV, Hepatitis B and C and CMV status. Future studies would be planned to address these issues and capture this information.

## Data Availability Statement

The datasets presented in this study can be found in online repositories. The names of the repository/repositories and accession number(s) can be found below: https://doi.org/10.6084/m9.figshare.19208328.v2.

## Ethics Statement

The studies involving human participants were reviewed and approved by the JIPMER Institute Ethics committee (Human studies), AIIMS Institute Ethics committee and PHE, UK (India Study Number JIP/IEC/2015/11/522, UK Study Number PHE0186). The patients/participants provided their written informed consent to participate in this study.

## Author Contributions

HG, KE, MH, NW, PP SP, and KK conducted the experimental work. JS, SK, HB, IA, SS, and NJ provided control and patient samples and clinical and scientific expertise to the project. KE, NJ, SV, PP, and KK designed the study protocol and managed the study. HG, KE and KK conducted the data analysis. HG, KE, NJ, SV, and KK wrote and edited the paper. All authors contributed to the article and approved the submitted version.

## Funding

This study was funded by the UK Department of Health and Social Care Grant in Aid and the Public Health England Pipeline fund. The views expressed in this publication are those of the authors and not necessarily those of PHE or the DH.

## Conflict of Interest

The authors declare that the research was conducted in the absence of any commercial or financial relationships that could be construed as a potential conflict of interest.

## Publisher’s Note

All claims expressed in this article are solely those of the authors and do not necessarily represent those of their affiliated organizations, or those of the publisher, the editors and the reviewers. Any product that may be evaluated in this article, or claim that may be made by its manufacturer, is not guaranteed or endorsed by the publisher.

## Supplementary Material

Supplementary Table 1 **Table S1.1**. Details of Patient and Control Samples **Table S1.2** Summary of normalised ELISA data for all samples included in the study **Table S1.3** Summary statistics for al ELISA data from the UK-CNTRL group **Table S1.4** Summary statistics for al ELISA data from the P-CNTRL group **Table S1.5** Summary statistics for al ELISA data from the A-CNTRL group **Table S1.6** Summary statistics for al ELISA data from the A-EPTB group **Table S1.7** Summary statistics for al ELISA data from the UK-CNTRL group **Table S1.8** Summary statistics for al ELISA data from the LTBI group **Table S1.9** Summary statistics for al ELISA data from the PTB group **Table S1.10** Summary of the mean, median and standard deviation biomarker expression values across all groups **Table S1.11** Summary of the biomarker expression paired t-test p-values across all groups Summary statistics for al ELISA data from the PTB group **Table S1.12** Summary of the average, fold-change and p-values for biomarker expression for the P-CNTRL, LTBI_PR and LTBI_NPR groups **Table S1.13** Correlation of biomarker target expression with QFT-GIT, T.SPOT and TST positivity in the combined LTBI group. **Table S1.14** Summary statistics for biomarker expression correlation matrices across all biomarkers and groups.

Supplementary Table 2 **Table S2.1** Random Forest modelling analysis for classification of Active TB and PTB from all combined control groups **Table S2.2** Single biomarker ROC curve analyses across all pairwise group combinations **Table S2.3** 5- and 3-biomarker panel ROC curve analyses across control and active TB disease groups. **Table S2.4** 5- and 3-biomarker panel ROC curve analyses across control and active TB disease groups and performance according to WHO triage test performance characteristics, plus positive and negative predictive values **S2.5** 5- and 3-biomarker panel ROC curve analyses across control and active TB disease groups and performance according to WHO confirmatory test performance characteristics, plus positive and negative predictive values.

Supplementary Information File S1 **Figure S1.1**. Average and standard deviation for biomarker expression across active TB and control groups; Δ UK-CNTRL, ■ P-CNTRL, □ A-CNTRL, ◊ A-EPTB, ♦ J-EPTB, ● PTB **Figure S1.2**. Average and standard deviation biomarker expression across active TB and control groups; ○ UK-CNTRL, ● A-CNTRL, ▲P-CNTRL, ■ LTBI_NPR, □ LTBI_PR.
