# Supplementary material for: Evaluation of Host Protein Biomarkers by ELISA From Whole Lysed Peripheral Blood for Development of Diagnostic Tests for Active Tuberculosis
Source: Front Immunol. 2022 May 20;13:854327. doi: 10.3389/fimmu.2022.854327 (PMC9205408; doi:10.3389/fimmu.2022.854327)
Supplement: Supplementary Information File S1 — Figure S1.1. Average and standard deviation for biomarker expression across active TB and control groups; Δ UK-CNTRL, ■ P-CNTRL, □ A-CNTRL, ◊ A-EPTB, ♦ J-EPTB, ● PTB Figure S1.2. Average and standard deviation biomarker expression across active TB and control groups; ○ UK-CNTRL, ● A-CNTRL, ▲P-CNTRL, ■ LTBI_NPR, □ LTBI_PR. [file Presentation_1.pptx]

## Slide 1
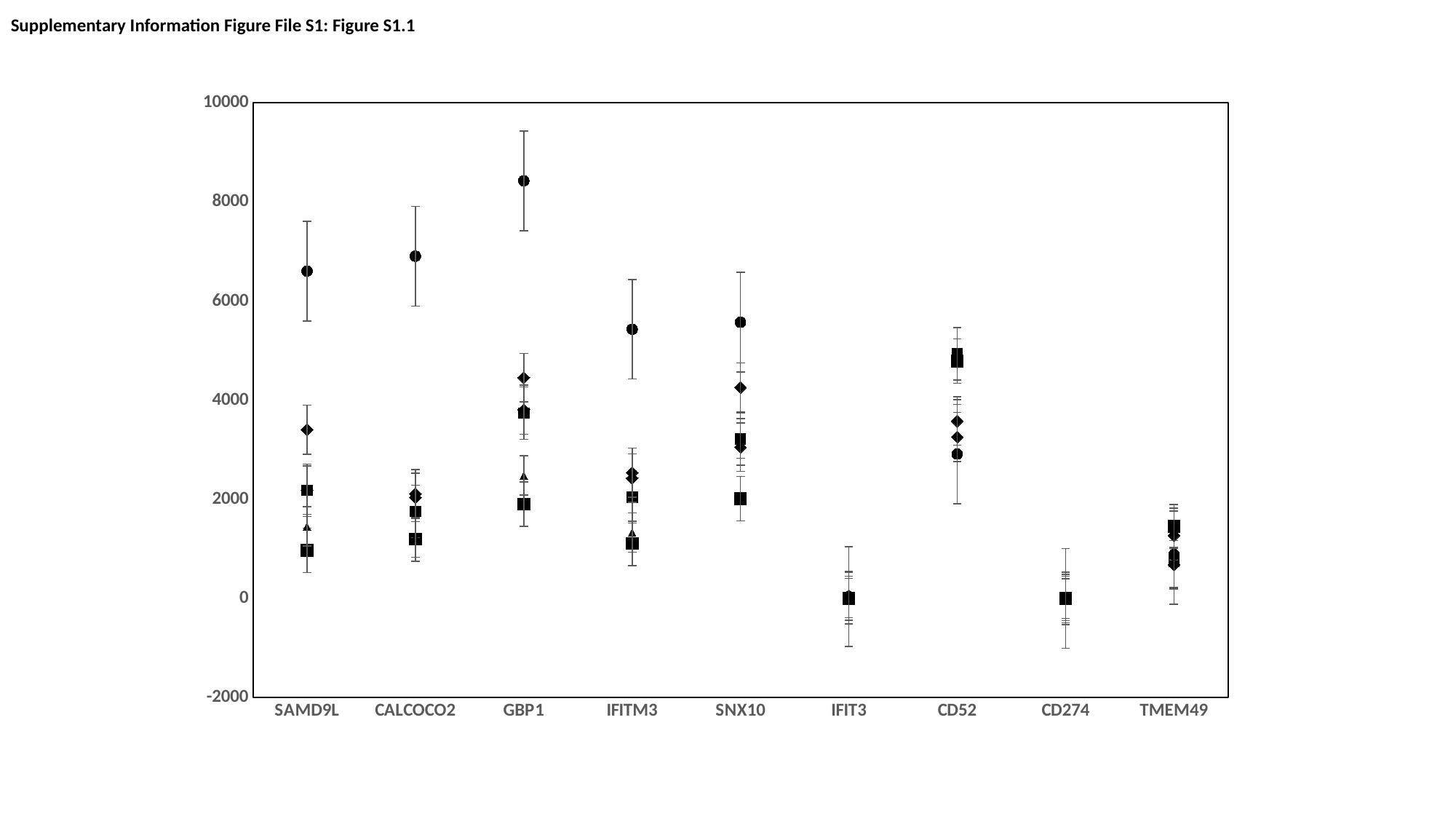

Supplementary Information Figure File S1: Figure S1.1
### Chart
| Category | P-CNTRL | A-CNTRL | UK-CNTRL | J-EPTB | A-EPTB | PTB |
|---|---|---|---|---|---|---|
| SAMD9L | 973.617866319108 | 2182.342553819331 | 1454.9875597804262 | 3407.271586879433 | 2188.2252637681163 | 6607.244029526704 |
| CALCOCO2 | 1201.194670100249 | 1759.3558917989421 | 1230.2033190893897 | 2111.026684207337 | 2039.7194557142857 | 6907.197204968943 |
| GBP1 | 1903.0698680713542 | 3742.859159916853 | 2483.8072983674983 | 3810.8349796096036 | 4458.629200709535 | 8428.46794370362 |
| IFITM3 | 1110.292014678899 | 2048.7362248 | 1332.2394166666663 | 2541.7912784090904 | 2428.40225862069 | 5433.962053571429 |
| SNX10 | 2017.8900205995394 | 3222.3423157894736 | 3230.3904244991386 | 4260.552122241087 | 3053.7941789473675 | 5574.266885964913 |
| IFIT3 | 0.0 | 20.026283520408168 | 9.61551076923077 | 57.79374952038369 | 45.09814150943398 | 41.23076530612246 |
| CD52 | 4791.396715843345 | 4939.62627594374 | 3616.096521188411 | 3260.9611523865133 | 3583.360212233334 | 2915.4634485871275 |
| CD274 | 0.09628086749851457 | 1.2695378521126768 | 0.06047348484848485 | 0.9951550000000008 | 1.1505165000000013 | 2.207866651336591 |
| TMEM49 | 1457.2103738529893 | 753.8413699276647 | 1425.5780657803607 | 1274.223712018793 | 682.5368908058554 | 888.5495068986485 |

## Slide 2
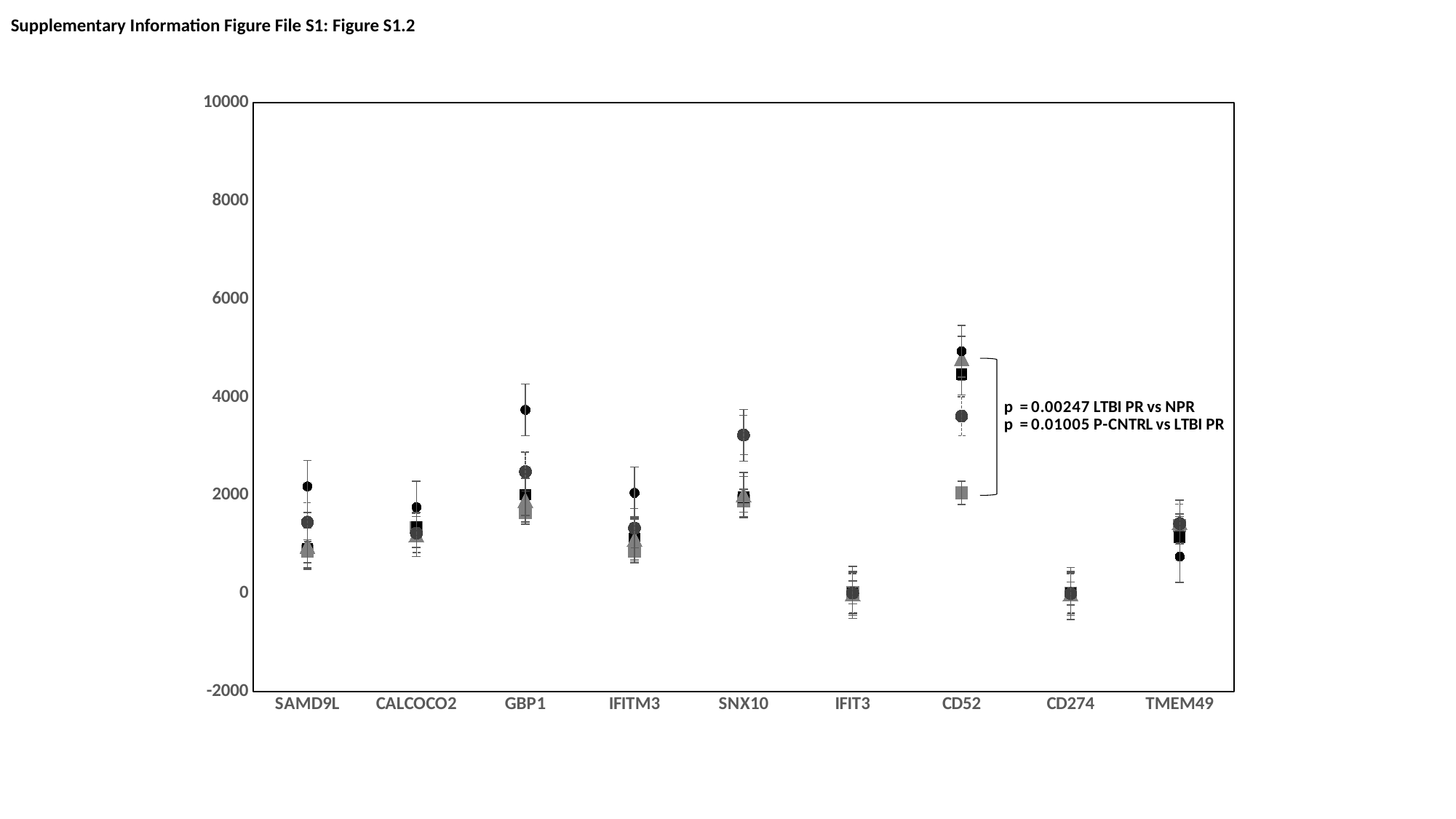

Supplementary Information Figure File S1: Figure S1.2
### Chart
| Category | PR AVE | NPR AVE | P-CNTRL | A-CNTRL | UK-CNTRL |
|---|---|---|---|---|---|
| SAMD9L | 860.9190774167281 | 918.2819013738907 | 973.617866319108 | 2182.342553819331 | 1454.9875597804262 |
| CALCOCO2 | 1336.6862104330637 | 1362.0853019897038 | 1201.194670100249 | 1759.3558917989421 | 1230.2033190893897 |
| GBP1 | 1651.4634946254516 | 2012.9056425007795 | 1903.0698680713542 | 3742.859159916853 | 2483.8072983674983 |
| IFITM3 | 858.7764255130865 | 1112.2477025100834 | 1110.292014678899 | 2048.7362248 | 1332.2394166666663 |
| SNX10 | 1889.4339485725295 | 1964.637353611841 | 2017.8900205995394 | 3222.3423157894736 | 3230.3904244991386 |
| IFIT3 | 24.826706043956044 | 5.878835426023254 | 0.0 | 20.026283520408168 | 9.61551076923077 |
| CD52 | 2050.710182550466 | 4471.776391000707 | 4791.396715843345 | 4939.62627594374 | 3616.096521188411 |
| CD274 | 0.025277777777777777 | 0.05655527336860671 | 0.09628086749851457 | 1.2695378521126768 | 0.06047348484848485 |
| TMEM49 | 1384.8348242098887 | 1149.6969696002448 | 1457.2103738529893 | 753.8413699276647 | 1425.5780657803607 |
